# Future Risks of Pest Species under Changing Climatic Conditions

**DOI:** 10.1371/journal.pone.0153237

**Published:** 2016-04-07

**Authors:** Lisa Biber-Freudenberger, Jasmin Ziemacki, Henri E. Z. Tonnang, Christian Borgemeister

**Affiliations:** 1 Center for Development Research (ZEF), Department of Ecology and Natural Resources Management, University of Bonn, Walter-Flex-Str. 3 53113, Bonn, Germany; 2 International Maize and Wheat Improvement Center (CIMMYT), ICRAF House, United Nations Avenue, Gigiri, P. O. Box 1041 Village Market, 00621, Nairobi, Kenya; Federal University of Viçosa, BRAZIL

## Abstract

Most agricultural pests are poikilothermic species expected to respond to climate change. Currently, they are a tremendous burden because of the high losses they inflict on crops and livestock. Smallholder farmers in developing countries of Africa are likely to suffer more under these changes than farmers in the developed world because more severe climatic changes are projected in these areas. African countries further have a lower ability to cope with impacts of climate change through the lack of suitable adapted management strategies and financial constraints. In this study we are predicting current and future habitat suitability under changing climatic conditions for *Tuta absoluta*, *Ceratitis cosyra*, and *Bactrocera invadens*, three important insect pests that are common across some parts of Africa and responsible for immense agricultural losses. We use presence records from different sources and bioclimatic variables to predict their habitat suitability using the maximum entropy modelling approach. We find that habitat suitability for *B*. *invadens*, *C*. *cosyra* and *T*. *absoluta* is partially increasing across the continent, especially in those areas already overlapping with or close to most suitable sites under current climate conditions. Assuming a habitat suitability at three different threshold levels we assessed where each species is likely to be present under future climatic conditions and if this is likely to have an impact on productive agricultural areas. Our results can be used by African policy makers, extensionists and farmers for agricultural adaptation measures to cope with the impacts of climate change.

## Introduction

The combination of about 795 Million people suffering from undernourishment [1] and an expected population increase from 7 up to 9 billion by 2050 is projected to lead to an increase in the need for food between 70–100% compared to 2010 [2]. At the same time, the United Nations have agreed to aim at reaching the Sustainable Development Goals (SDGs) that include the eradication of hunger and the sustainable use of terrestrial ecosystems. Yet, it is very likely that competition for the limited arable land will increase under these, at least partially, conflicting goals and that efficient and sustainable use of already cultivated land will become even more important in the future than it is already today.

The global scale of climate change (CC) and the necessity for sustainable poverty mitigation strategies increases the need to quantify CC impacts. Especially the effects on agricultural production are of major importance for the local populations, in particular famers, national governments, regional bodies like the African Union (AU), international development partners and other stakeholders [3]. Such data can increase awareness and help to develop coping strategies for the vulnerable, as well as enable them to adapt better to a changing environment. It would also allow national governments to design and implement changes in policies, necessary for mitigating the effects of climate change [4].

In Africa, agriculture is the source of income for many families and represents over two thirds of livelihoods of the poor. About 65% of full-time employment is in the agricultural sector and over half of the total export earnings derive from agricultural goods [5]. Large losses of agricultural production can be attributed to pests. In Africa alone 12.8 billion US$ were estimated to be lost to pathogens, insects and weeds between 1988 and 1990. Insects are the economically most relevant pest group and the cause for about 1/3 of the actual crop production equal to 4.4 billion US$ being lost [6]. Many people in sub-Saharan Africa (SSA) heavily rely on natural resources and have a relatively low tolerance towards climatic and economic stress because of high poverty levels and lack of alternative sources of income [4]. The often limited capacity to adapt to changes through a lack of knowledge and education, further increases the vulnerability of Africa´s poor to CC impacts [7]. Apart from the more general negative effects that CC is expected to have on agricultural production in many developing countries, it is also likely to have a profound impact on the abundance and distribution of many pest species.

CC impacts are expected for the whole planet, but farmers in the developed world are likely to be better prepared to deal with potentially increasing numbers of pests or invasive species than those in less developed countries. Main reasons for this include greater financial means of farmers in the developed world to utlize different pest control strategies, be they biological, synthetic pesticides or genetically modified crops, interventions that are most often economically not feasible for most of the producers in the developing world. By consequence, this then results in greater food insecuirty in these regions.

Agricultural productivity strongly depends on continued innovations to control pests as they develop resistances to different control measures, such as synthetic pesticides, or disperse to new regions [2]. While most studies estimate increasing numbers and distribution for many pest species, responses of individual species may vary depending on, among others, the bioclimatic conditions under different CC scenarios. In this study we use species distribution modelling to evaluate the possible extent and change of the habitat suitability across Africa for three important pest species, *Tuta absoluta*, *Ceratitis cosyra* and *Bactrocera invadens*, under future CC.

*Tuta absoluta* (Meyrick) (Lepidoptera: Gelechiidae), also called the tomato leaf miner, is a key threat to European and African tomato production. The pest originates from South America and is spreading rapidly over southern Europe into northern Africa since its first detection in Spain in 2006. Its high reproductive capacity and rapid development of resistance to many different insecticides make conventional chemical control very challenging. Consequently yield losses of 80–100% have been reported [8]. *Ceratitis cosyra* (Walker) (Diptera: Tephritidae), or mango fruit fly, is a serious pest in smallholder and commercial mango plantations across SSA. It is native to afrotropical regions and is commonly intercepted in Europe as larvae in infested mangoes [9]. *Bactrocera invadens* (Drew, Tsura and White, 2005) (Diptera: Tephritidae) is a member of the oriental fruit fly species complex, possibly of Sri Lankan origin, and since 2003 has spread across East and West Africa. It has a very broad host range and feeds on a wide variety of unrelated wild and cultivated crops. Due to its highly destructive and invasive potential, *B*. *invadens* has become economically the most important fruit fly in Africa [10].

Many studies investigating the impact of CC on pest distribution show increasing densities or an expansion of the geographical range of pests [11–14]. Significant research focusing on the possible impacts of CC on insects has already been carried out in temperate areas of the world [15–17]. It was reported that warmer winters might advance the survival rate of insects and permit a faster population revival that will consequently built-up in spring [17]. Additionally, an increase in the length of the cultivating period is projected, permitting multivoltine species to generate a higher number of generations per year and therefore upsurge invasions by alien species [15,18].

In comparison to temperate areas, tropical countries of Africa are highly disposed to insect pest problems and outbreaks due to their year-round favorable environments for insects’ population growth and host plants availability [19]. Further studies revealed that warming in tropical areas, although tiny in scale, is expected to yield harmful consequences because tropical insects are very sensitive to small changes on the magnitude of climatic veriables, such as temperature [20,21].

Species distribution modelling with presence-only data can be used to model the current habitat suitability of a species and project future suitabilities under changed climatic conditions. Models of this type have already been developed for several pest species in Asia [22–24], North America [25,26], several European countries [27], as well as for the global distribution of pests [28–30]. Fewer modelling approaches have been carried out with a focus on Africa [31,32]. Considering the importance of agriculture for the continent, more studies assessing pest species dispersal are needed to estimate potential future yield losses as a result of CC.

Climate change is likely to have negative impacts on food security and livelihoods of farmers in Africa through change of the number of generations and distributions of pest species [13,33,34]. Despite this, there are only few studies providing specific information on which species are likely to affect which regions under CC in Africa. This information, however, is vital for farmers, if they want to adapt to the impacts of CC.

## Methods

### Pest species and presence records

**Fig 1:**
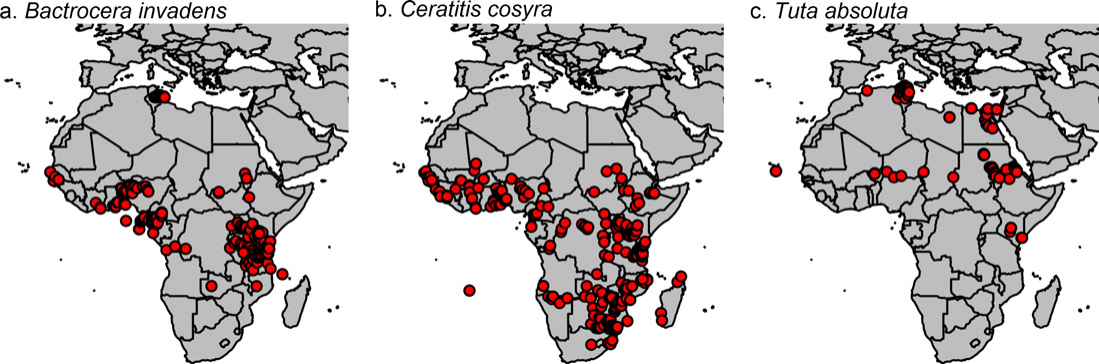
Presence records for 3 important pest species accessed through GBIF and other literature (see Table 1 for an overview).

Presence records of the three important insect pest species were used from different sources including the Global Biodiversity Information Facility (GBIF, www.gbif.org) and published studies (Table 1, Fig 1).

**Table 1:** Species names, source of presence records, date of data access, total number of presence records found for Africa in the original source and number of presence records used for training and testing.

Species Name	Source	Access Date	No. of presence records		
			from source	used for training	used for testing
*Tuta absoluta*	[35–40]	03/02/15	542	141	60
*Ceratitis cosyra*	[35,41,42]	13/02/15	595	175	75
*Bactrocera invadens*	[10,31,43]	05/01/15	226	118	50

Presence data were randomly split in two sets for model training (70%) and testing (30%). A number of presence records had to be excluded because of missing environmental information or other issues with the geographical coordinates (e.g. coordinates pointed towards locations in the ocean). All used presence records are provided in S1 File.

### Environmental variables

Presence records (Fig 1) were used in combination with bioclimatic variables downloaded from the WorldClim database ([44] accessed through [45]) to assess current and future habitat suitability of the pest species using maximum entropy modelling. Current climatic data correspond to interpolations of observed data from 1950 to 2000, while future climatic conditions represent downscaled modelled data averaged for the years 2041 to 2060 according to the IPCC5 (CMIP5). Three different global circulation models (GCMs) and four representative concentration pathways (RCP) were used in a 2.5 arc minute (4.6 km) resolution.

Not all of the selected GCMs provide data for all RCPs (Table 2). The GCMs were selected based on their ability to reflect the dynamics of the West African monsoon [46,47] and their representation of modelled precipitation and temperature values from different CMIP5 models [48]. While the GFDL model represent colder and wetter values, the HadGEM model values are warmer and drier and the MPI-ESM values are close to the multi-model mean.

Habitat suitability was modelled using bioclimatic variables under each GCM and RCP.

**Table 2:** Overview of global circulation models (GCMs) and representative concentration pathways (RCPs) that were available and accessed via the database WordlCim ([44] accessed through [45]).

	RCP 26	RCP 45	RCP 60	RCP 85
GFDL-ESM2G	•	•	•	
HadGEM-ES	•	•	•	•
MPI-ESMLR	•	•		•

Bioclimatic variables are calculated based on monthly values of temperature and rainfall but are likely to be biologically more meaningful than simple average values since they represent both annual trends but also seasonality and extreme conditions (Table 3).

**Table 3:** Overview of bioclimatic variables used for species distribution modelling.

Abbrev.	Bioclimatic Variable Description
BIO1	Annual Mean Temperature
BIO2	Mean Diurnal Range (Mean of monthly (max temp—min temp))
BIO3	Isothermality (BIO2/BIO7) (* 100)
BIO4	Temperature Seasonality (standard deviation *100)
BIO5	Max Temperature of Warmest Month
BIO6	Min Temperature of Coldest Month
BIO7	Temperature Annual Range (BIO5-BIO6)
BIO8	Mean Temperature of Wettest Quarter
BIO9	Mean Temperature of Driest Quarter
BIO10	Mean Temperature of Warmest Quarter
BIO11	Mean Temperature of Coldest Quarter
BIO12	Annual Precipitation
BIO13	Precipitation of Wettest Month
BIO14	Precipitation of Driest Month
BIO15	Precipitation Seasonality (Coefficient of Variation)
BIO16	Precipitation of Wettest Quarter
BIO17	Precipitation of Driest Quarter
BIO18	Precipitation of Warmest Quarter
BIO19	Precipitation of Coldest Quarter

Mean values of future habitat suitability were calculated over all modelled habitat suitability datasets. They were calculated first over all models for all RCPs before they were calculated over all models. Therefore the lack of data for two RCPs for two GCMs only results in a lower confidence level for the results of the two higher RCPs. The overall results, however, are not skewed towards higher or lower emission scenarios.

### Modelling approach and evaluation

The machine learning approach Maxent (www.cs.princeton.edu/~schapire/maxent/) was used to assess habitat suitability based on maximum entropy. Maxent has been shown to perform particularly well for modelling presence-only data [49]. Maxent is used to predict the environmental suitability for the species as a function of the given environmental variables. Hereby the distribution probability is estimated by finding the distribution of maximum entropy that satisfies a set of constraints from environmental variables. The results serve as an approximation of a species’ ecological niche under the studied environmental conditions [50]. Maxent belongs to the family of models relying solely on presence records of the investigated species.

Environmental variables were reduced to only the three most important variables, with the highest variable contributions calculated according to a first model run based on all variables.

A bias file was produced with the package kernSmooth in R [51] to correct for uneven distributions of sampling efforts across the study area. For this purpose coordinates from presence 18,108,111 records of all animals from GBIF were used to create a kernel density estimate map across Africa with a bandwith of 5 in each direction. Since the sample size is very large, we chose a relatively small bandwidth which is still large enough to result in values greater than 0 in each pixel cell.

The performance of the model is characterized by the area under the curve (AUC). The AUC is obtained by the threshold independent receiver operating characteristic (ROC) analysis [50]. In the process of modelling, 70% of occurrence localities were randomly selected as training data, while the remaining 30% served for testing the resulting models. The ROC method is based on the AUC when model sensitivity is plotted against 1 minus model specificity. This method has been shown to be effective in evaluating model performance and being independent of prevalence compared to the more commonly used kappa statistic [52–54].

The output of the model represents an area with conditions comparable to those in the species’ known occurrence range, whereas the values between 0 and 1 indicate regions with no or most suitable habitat conditions, respectively.

The response of the species distribution model to specific environmental variables was investigated through the permutation importance and response curves of each bioclimatic variable. The permutation importance is calculated based on the drop or increase of the AUC, when the respective environmental variable is altered.

Based on a threshold value, habitat suitability can be converted from probability maps to species distribution maps. In this study we used three different threshold values to create distribution maps and overlaid them with agricultural production intensity with current and future species presence polygons. We chose to use three commonly used threshold levels to display the variability in distribution maps dependent on the selected threshold level (Table 4). The agricultural intensity map is an extract of the data on global crop land published by Ramankutty et al. [55] and represents the fraction of area being under use as cropland for each raster cell at a resolution of 5 min (10 km).

**Table 4:** Thresholds used to estimate species distribution maps for all three species. The "Balance" threshold minimizes 6 * training omission rate + .04 * cumulative threshold + 1.6 * fractional predicted area.

	*B*. *invadens*	*C*. *cosyra*	*T*. *absoluta*
Equal training sensitivity and specificity	0.266	0.378	0.379
Maximum training sensitivity plus specificity	0.196	0.187	0.463
Balance training omission, predicted area and threshold value	0.063	0.095	0.088

All maps are displayed using a simple Plate Carrée (WGS 84) projection. This projection is an equidistant projection, which was chosen as it is appropriate for large-scale studies to balance area distortion and shape.

## Results

### Statistical model evaluation

AUC values for training data of all species are >0.85 and AUC values for test data of all species are >0.8 (Table 5). This demonstrates that all models show a good predictive performance.

**Table 5:** Area under curve (AUC) values of training and test data as an evaluation parameter of model performance for all investigated species.

Species	AUC training data	AUC test data
*Bactrocera invadens*	0.929	0.917
*Ceratitis cosyra*	0.874	0.861
*Tuta absoluta*	0.938	0.868

### Habitat suitability under current and future climatic conditions

**Fig 2:**
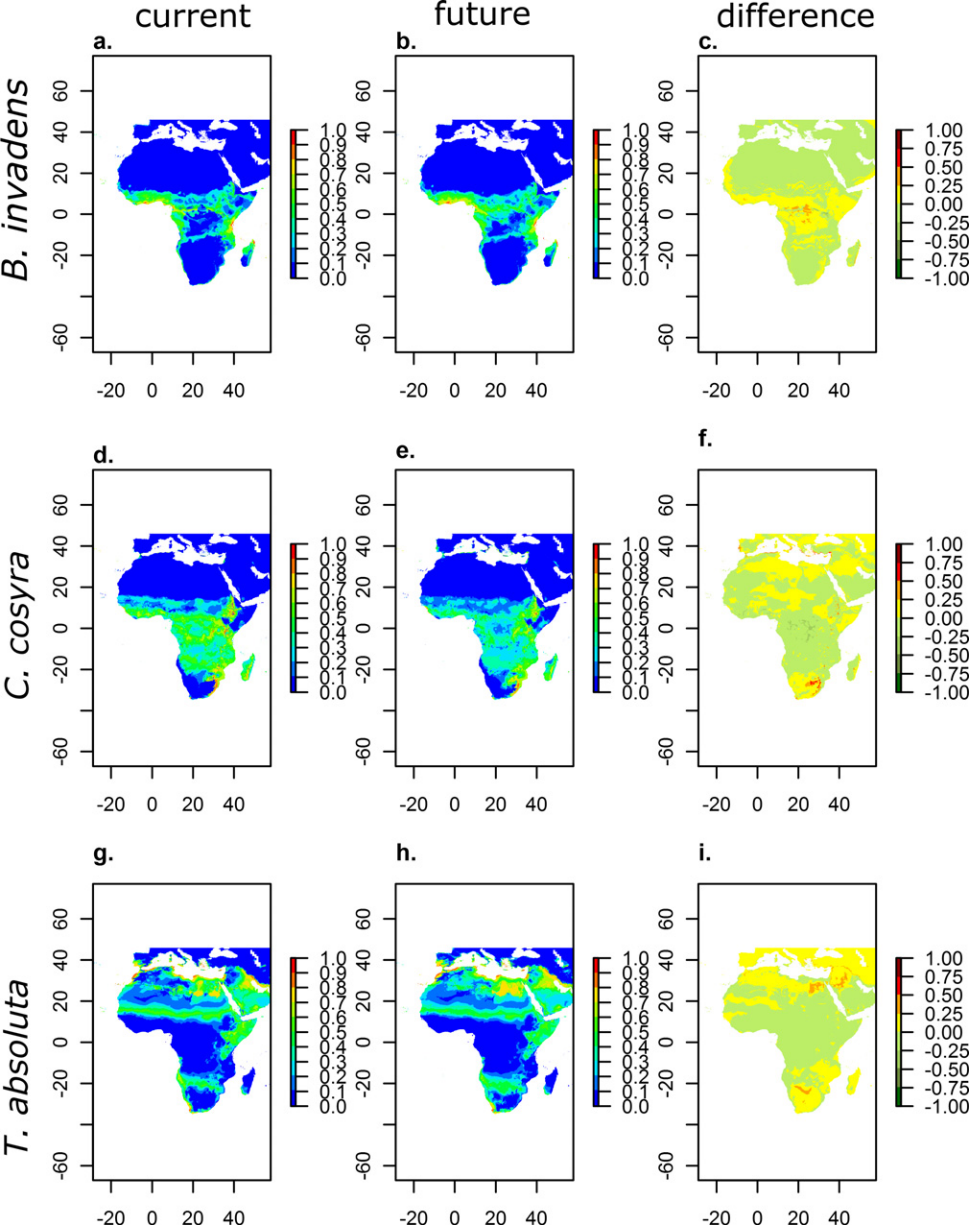
Habitat suitability under current and future climatic conditions as well as change of habitat suitability of *Bactrocera invadens* (a-c), *Ceratitis cosyra* (d-f), and *Tuta absoluta* (g-i) modelled as logistic outputs of Maxent.

Current habitat suitability shows which area each species is likely to inhabit under current climatic conditions (Fig 2).

*Bactrocera invadens* (Fig 2A) and *C*. *cosyra* (Fig 2D) show high values of habitat suitability scattered across SSA. However, while *C*. *cosyra* seems to be able to inhabit also Central Africa, *B*. *invadens* is more restricted to the coastal areas. Suitable habitats for *T*. *absoluta* (Fig 2G) are located almost exclusively in North Africa (Algeria, Libya and Egypt), across the Sahelzone, and on the Arabian Peninsula. Especially in Egypt tomato, the host plant of *T*. *absoluta*, is an important cash crop. Egypt belongs to the top tomato producing countries worldwide (http://faostat.fao.org/). Therefore, we expect large negative economic impacts of *T*. *absoluta* for tomato producers under future CC.

The shown values represent mean values over model outputs with all available 10 bioclimatic variable datasets (3 GCMs and 4 RCPs, Table 2) using the three most important variables. Mean values over each RCP can be found for each species in S1–S3 Figs. Future habitat suitability of the three species appears to be overall similar to the current suitability (Fig 2). Although species react differently to projected climatic changes, in this study all three species showed, at least for parts of Africa, an increase in habitat suitability. Across large parts of the continent they show an either unchanged or even slightly increasing number of suitable habitat sites. Within the maps highlighting the change of habitat suitability an increase of suitability is indicated by the colours yellow to red, while the suitability of areas with the colours light-green to dark-green is likely to decrease.

### Environmental variable importance and impact

Permutation importance varies between species and for all environmental variables (Table 6). Response curves of the models for the three most important environmental variables of each species are shown in S4 Fig. For *B*. *invadens* temperature seasonality (BIO4), temperature annual range (BIO7) and precipitation of the driest quarter (BIO17) are the most important variables. The species is preferring for all of these variables rather low values. Especially temperature seasonality and annual range are unlikely to change dramatically in the future, which is probably the reason why *B*. *invadens* does not respond strongly to changing climatic conditions.

**Table 6:** Permutation importance as drop in area under curve (AUC) after values of variables on training and presence data had been randomly permuted for each environmental variable in turn; values represent normalized percentage values based on a first model run including all available bioclimatic variables (values before slash) and a second model run including only the three most important variables (values after slash) according to the first model run.

Variable Abbrev.	Permutation importance (%)		
	*Bactrocera invadens*	*Ceratitis cosyra*	*Tuta absoluta*
BIO1	0.5	0	2.3
BIO2	1.6	0.7	4.9
BIO3	0.3	15	1.6
BIO4	**14/63.3**	6.4	0
BIO5	0	0.4	0
BIO6	3.4	0	**28.9/31.2**
BIO7	**15.3/23.4**	5.5	1.4
BIO8	4.4	8.1	**8/20.8**
BIO9	0.4	0.3	3.8
BIO10	0.5	1.4	1.2
BIO11	1.3	**11.1/28.5**	0
BIO12	13.5	**8.8**	**40.5/48**
BIO13	5.2	5.3	6
BIO14	0.5	**8.9/13.2**	1.2
BIO15	5.1	5.9	1.3
BIO16	4.2	**10.2/58.3**	0
BIO17	**20.9/13.4**	4.8	0.6
BIO18	3.5	2.2	3.8
BIO19	4.8	5	4.5

The *C*. *cosyra* modelled suitability depends mainly on the mean temperature of the coldest quarter (BIO11), precipitation of the driest month (BIO14) and the precipitation of the wettest quarter (BIO16). Overall it prefers medium to high precipitation rates and temperatures >10°C throughout the year. Climate change projections indicate that in those areas of Africa, where we find increasing habitat suitability especially temperatures and precipitation are likely to increase under future CC. For *T*. *absoluta* the minimum temperature of the coldest month (BIO6), the mean temperature of the wettest quarter (BIO8) and annual precipitation (BIO12) are the most important parameters for suitable habitat conditions. The model indicates that it prefers higher temperatures and lower preciptition rates. Under CC more areas are projected to fulfill these requirements, especially in northern Africa.

### Pest impact on agricultural areas

**Fig 3:**
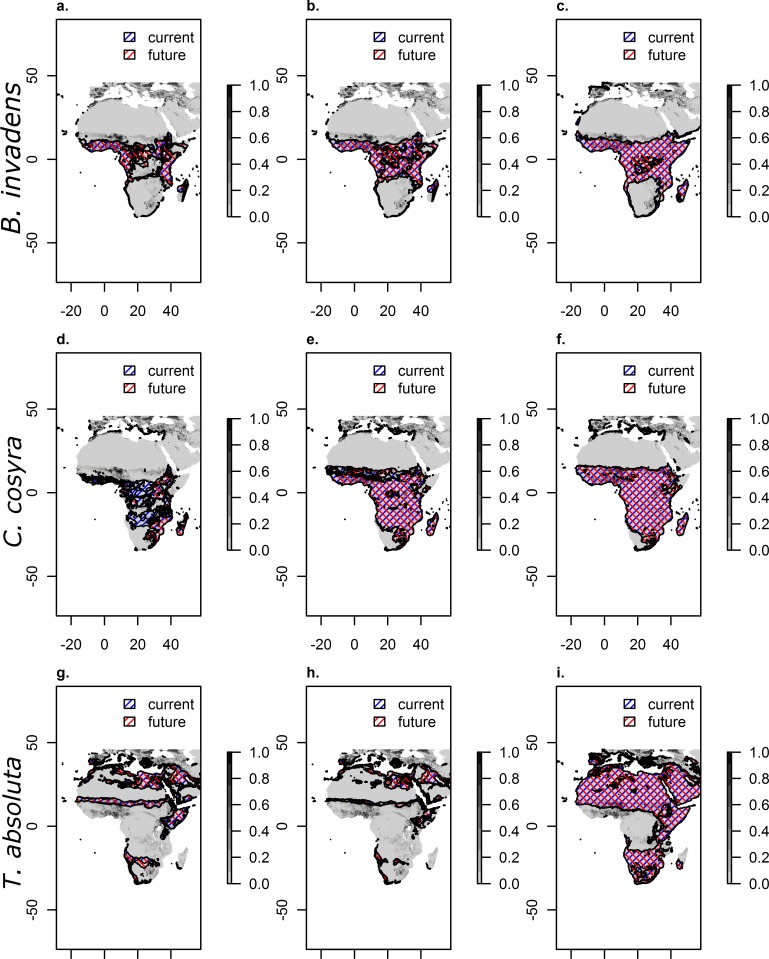
**Agricultural crop intensity overlaid with presence of each species under current and future climate assuming three different habitat suitability levels (Table 4): Equal training sensitivity and specificity (a-c), maxium training sensitivity plus specificity (d-f) and balancing training omission, predicted area and threshold value (g-i).** Agricultural crop intensity is reprinted from Ramankutty et al. [55] under a CC BY license, with permission from John Wiley and Sons, original copyright 2008.

Assuming a threshold habitat suitability at different level shows that although habitat suitability in some areas might be increasing, under the suitability threshold equal training sensitivity and specificity the species is still not predicted to be present. However, under a threshold value of maximum training sensitivity plus specificity as well as balancing training omission, predicted area and threshold value much larger proportions of Africa are predicted to be inhabited by the species (Fig 3).

Comparing the areas where the species is predicted to be present under current and future climate shows that *B*. *invadens* and *T*. *absoluta* are unlikely to shift their habitat at all. For all three applied habitat suitability thresholds species distribution does not change under current and future climatic conditions even though habitat suitability generally is increasing over large parts of the continent (compare with Fig 2). For *C*. *cosyra* we find a decreasing number of areas in southern and Central Africa being inhabited under a higher threshold (Fig 3D). For the two higher thresholds mostly the same areas are being predicted as suitable under future as under current climatic conditions.

Comparing current and future distributions of the studied species with agricultural crop intensity indicates that those areas with high agricultural production are not under a higher threat under future CC than under current conditions. Habitat extent of all tested species is likely to remain constant, shift to less productive sites or decrease.

**Fig 4:**
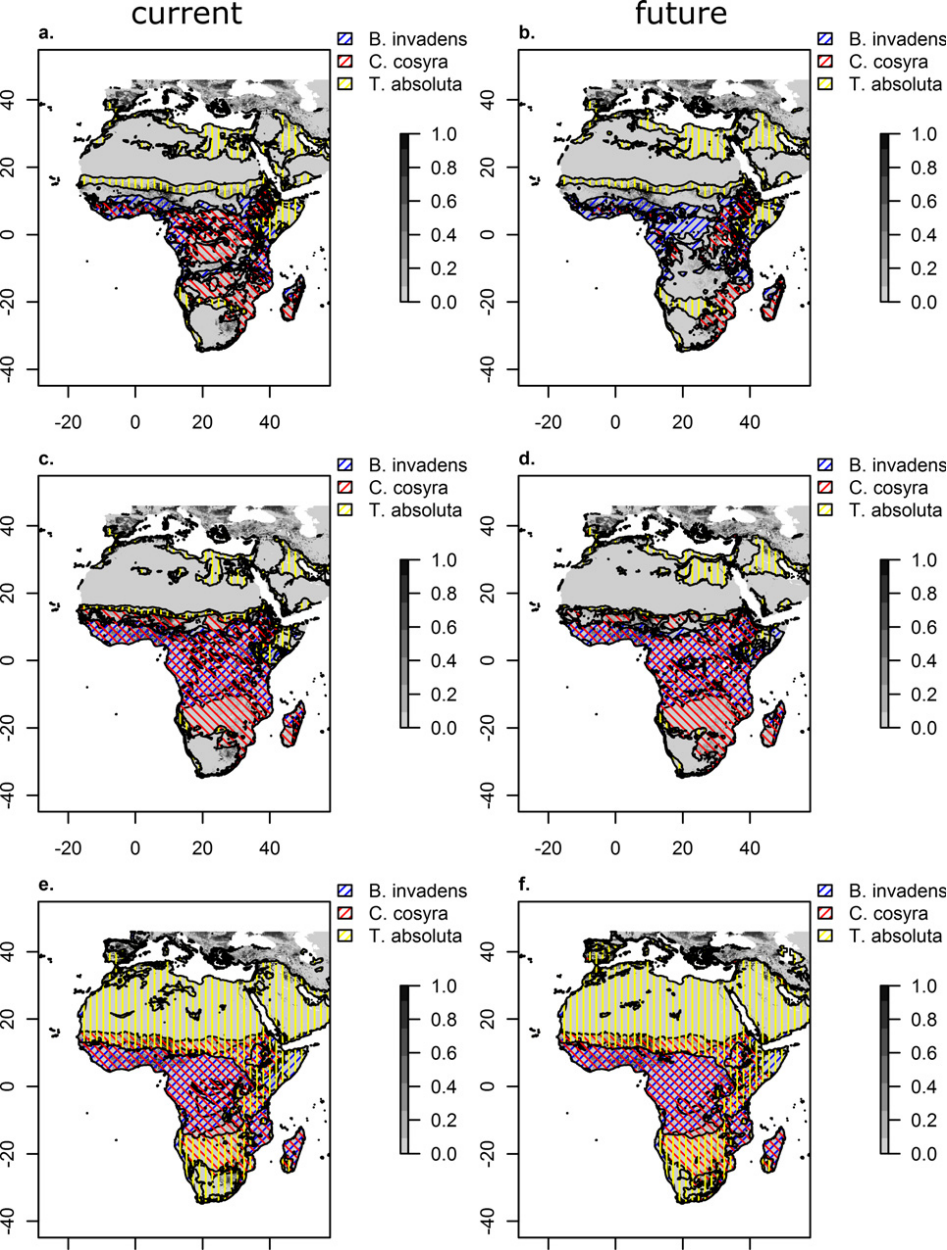
**Agricultural crop intensity overlaid with presence of all three species combined under current and future climate assuming three different habitat suitability levels (Table 4): Equal training sensitivity and specificity (a and b), maxium training sensitivity plus specificity (c and d) and balancing training omission, predicted area and threshold value (e and f).** Agricultural crop intensity is reprinted from Ramankutty et al. [55] under a CC BY license, with permission from John Wiley and Sons, original copyright 2008.

Overlaying the habitat of all three species for all three thresholds under current and future climate shows that especially for lower threshold levels CC impact seems to be deniable (Fig 4). Under these levels almost all areas are already under current conditions affected by at least one of the pest species (Fig 4E and 4F). For higher threshold levels, CC seems to have a rather positive impact since the distribution of *C*. *cosyra* is slightly decreasing, while the distributions of the two other species remain largly constant (Fig 4A and 4B).

## Discussion

In this study we assessed the impact of CC on three important agricultural pests of different crops in Africa. We used environmental variables to assess CC effects in combination with different presence records obtained from multiple sources. Few other studies have investigated the distribution and potential for dispersal of pests in Africa [31,32]. Here we modelled the future distribution of three important pests in Africa under current and future climatic conditions. Area under the curve statistics showed high values for all species confirming a good model performance.

Climate Change impacts on agriculture poses a major threat to agricultural productivity in Africa [34,56,57]. Even without consideration of the impact of pests under CC decreasing yields for major crops between 5% for maize and 17% for wheat have been projected by 2050 across the entire African continent [56]. At the same time many studies indicate that productivity could also benefit under CC if suitable adaptation measures are implemented [58]. Adaptation through better agricultural management as well as decision making under consideration of CC risks, as suggested for example by Vermeulen et al. [59], is strongly influenced by the availability of information on CC impacts, such as the future distribution of important pests.

Our results show that species presence or absence depends strongly on the choice of a habitat suitability threshold. While in a certain area a species might not be predicted to be present under a higher threshold level, it might yet be under a lower threshold level. Using variable threshold levels, as we did in this study, shows this uncertainty, which can also be translated to risk levels of pest species invasion. Such information are useful for farmers, NGOs and policy makers as they give them a prioritisation list on which pest species to focus on first and which species are of lesser concern to them. Furthermore, it provides a guideline which crops are recommendable to be planted and which should be avoided, if risks are to be minimized.

Presence-only data models stand beside those based on presence and absence data usually obtained through systematic sampling, e.g. generalized linear or additive models. However, presence data are often easier to obtain than verified absence data for example from databases and museum collections. Therefore, presence-only models, such as Maxent, GARP (Genetic Algorithm for Rule-Set Production) or ENFA (Ecological Niche Factor Analysis) are usually used for predictions based on presence-only data. GARP models are based on the integration or rejection of rules that are being tested to improve or decrease the predictive performance of the model. ENFA based predictions are calculated from uncorrelated factors explaining the differences between the whole study area and the area inhabited by the species. Maxent, on the other hand, assumes that a species distribution would follow a maximum entropy without any environmental constraints. The model predicts habitat suitability by fitting a probability distribution for the occurrence of the species across the whole area. Based on data from the different environmental variables different constraints are being formulated and considered in the model. According to to Elith et al. [49] Maxent performs relatively well compared to other presence-only models. However, Maxent also seems to suffer from a higher tendency of overfitting at low threshold levels than e.g. GARP models [60]. For this reason we used three different threshold levels to display species distribution maps.

Comparing our results with other studies shows some discrepancies in the predictions of habitat suitability for individual species under current and projected climate. For instance Tonnang et al. [30] modelled worldwide habitat suitability for *T*. *absoluta* using CLIMEX and found much larger areas of high suitability, especially across Central, eastern and southern Africa than in our study. De Meyer et al. [31] modelled habitat suitability for *B*. *invadens* in Asia, Africa and worldwide using two different approaches, i.e. GARP and Maxent with presence records from India, Sri Lanka and Bhutan. The Maxent modelling approach showed a much smaller area than the GARP approach. Suitable areas as predicted by De Meyer et al. [31] show some overlaps with highly suitable areas identified in our study. Yet for Central Africa we predicted lower suitability values than De Meyer et al. [31].

The discrepancies between the findings in our study and other publications may be the result from the level of uncertainty of methodologies currently used for species distribution modelling as well as different modelling input data. For example both, Tonnang et al. [30] and De Meyer et al [31] did not correct for sampling bias.

Another important fact is that the two species, *B*. *invadens* and *T*. *absoluta*, for which our modelling outcomes differ from those in other studies, are alien invasive species (AIS) in Africa. In Tonnang et al. [30] and De Meyer et al [31], both authors reported to have used records from the species native areas to develop their models; the obtained parameters were then projected to Africa for estimating areas of habitat suitability of the species. This approach differs from our modelling method, where we used only presence records from Africa. Being alien to Africa, the two species are likely to be still spreading and adapting to new environmental conditions to establish their final realized niches. When a species invades a new region its dispersal into new areas depends on environmental conditions and its ability to adapt can take a considerable amount of time. Hence, for model development using data of an AIS´ home might not be ideal for correctly predicting habitat suitability in a newly colonised region/area. Alternatively, native presence records of a species can be used to predict habitat suitability in different regions. Yet for species with high tolerance towards different environmental conditions this may also lead to lower predicted habitat suitability, especially in areas with environmental conditions not present in the species native area. If a species has for example a high tolerance to lower temperatures but presence points of areas with low temperatures were not included in the model because the species has not yet invaded such an area, predicted habitat suitability solely based on native records could be lower than the true suitability.

We used bioclimatic variables to characterize environmental conditions. However, other important environmental variables, such as soil properties, land cover and agricultural management interventions (e.g. use of pesticides or fertilizers) can influence species distribution but were not considered in this study. Including such variables has been described as a key challenge for modelling approaches [61]. While we agree that these variables are key to assess the final distribution of species, in this study, however, we emphasize on the necessity to assess only CC impact on three important agricultural pests in Africa. Since their distribution very much depends on which crops are grown where and how they are managed, projections under future climatic conditions are substantially influenced by individual decisions of farmers as well as market developments and thus difficult to model. Hence we decided to focus on assessing CC effects on the habitat suitability only.

In our study we used three different habitat suitability levels as an estimated threshold for species presence. This way we accounted for uncertainties related to the choice of a fixed threshold level. However, estimating species distributions from current and future habitat suitability is hampered by the fact that a species fundamental niche may be different from its realized niche, for example due to competition with other species or because of the effects the species itself has on its environment [62,63]. Furthermore, it should also be considered that simple presence of a pest species might not be harmful for agriculture if the density remains low. Here, we only looked at habitat suitability and the likelihood of presence or absence of a species. Nevertheless, it is reasonable to assume that higher densities are likely to occur if habitat suitability is high as long as the host species is present.

Furthermore, we were only using agricultural crop intensity as an estimation of agricultural productivity. Yet, areas which are currently not in use and/or less productive, might become so under future CC. Since pest species are highly dependent on their host species and agricultural production, this is likely to affect species distribution in a significant way. This aspect was, however, not considered in this study due to the lack of specific data.

Future research should include not only more important pest species in Africa, but also other environmental variables, and individual pest species should be linked to the cultivated areas of their respective host plants. Estimations of the impacts of pest species on agricultural production under projected CC would benefit from more species presence records as well as more detailed current and forecasted maps of crop production areas across the continent.

We believe that the results of this study can help policy makers, extension organisations and farmers to make adapted agricultural management decisions today while anticipating future CC impacts, for instance by choosing crops that are less susceptible to certain pest species. This can help to secure food production and livelihoods of farmers in the coming decades, when some pest species are likely to expand their distribution under CC. The maps can give farmers an orientation which crops are less likely to suffer under pests in the future and which they should avoid planting because of their association with higher risk of infestation. Using species distribution maps and climate scenarios and integrating them into land management decision systems can help to increase agricultural productivity, mitigate global hunger and thereby decrease competition for arable land.

## Supporting Information

S1 Fig Modelled habitat suitability for *Bactrocera invadens* for current climatic conditions (a) and future climatic conditions (b -e) as mean values over suitability modelled from bioclimatic data for 3 different Generalized Circulation Models (GCM) under each representative concentration pathways (RCP) scenario as well as mean change over all RCP scenarios.
(PDF)

S2 Fig Modelled habitat suitability for *Ceratitis cosyra* for current climatic conditions (a) and future climatic conditions (b—e) as mean values over suitability modelled from bioclimatic data for 3 different Generalized Circulation Models (GCM) under each representative concentration pathways (RCP) scenario as well as mean change over all RCP scenarios.
(PDF)

S3 Fig Modelled habitat suitability for *Tuta absoluta* for current climatic conditions (a) and future climatic conditions (b—e) as mean values over suitability modelled from bioclimatic data for 3 different Generalized Circulation Models (GCM) under each representative concentration pathways (RCP) scenario as well as mean change over all RCP scenarios.
(PDF)

S4 Fig Response curves of environmental variables indicating their effect on the predicted habitat suitability of Maxent with the logistic model output on the y-axis and the environmental variable on the x-axis. Response curves show effect of each variable in isolation on the model which makes interpretation of strongly correlated variables easier.
(PDF)

S1 File Presence records of *Bacterocera invadens* (Dataset A), *Ceratitis cosyra* (Dataset B) and *Tuta absoluta* (Dataset C) used in this study for distribution modelling.
(ZIP)

## References

[1] FAO. The State of Food Insecurity in the World 2015 [Internet]. 2015. Available: http://www.fao.org/hunger/key-messages/en/

[2] Godfray HCJ, Beddington JR, Crute IR, Haddad L, Lawrence D, Muir JF, et al Food Security: The Challenge of Feeding 9 Billion People. Science. 2010;327: 812–818. 10.1126/science.1185383 20110467

[3] Thornton PK, Jones PG, Alagarswamy G, Andresen J. Spatial variation of crop yield response to climate change in East Africa. Glob Environ Change. 2009;19: 54–65. 10.1016/j.gloenvcha.2008.08.005

[4] Thornton PK, Jones PG, Owiyo T, Kruska RL, Herrero M, Kristjanson P, et al. Mapping climate vulnerability and poverty in Africa [Internet]. ILRI; 2006 May. Available: https://cgspace.cgiar.org/handle/10568/2307

[5] Pretty J, Toulmin C, Williams S. Sustainable intensification in African agriculture. Int J Agric Sustain. 2011;9: 5–24. 10.3763/ijas.2010.0583

[6] Oerke E-C, editor. Crop production and crop protection: estimated losses in major food and cash crops Amsterdam ; New York: Elsevier; 1994.

[7] DFID research strategy 2008–2013, Working Paper Series: Sustainable Agriculture. Department for International Development (DFID); 2008 p. 27.

[8] Desneux N, Wajnberg E, Wyckhuys KAG, Burgio G, Arpaia S, Narváez-Vasquez CA, et al Biological invasion of European tomato crops by Tuta absoluta: ecology, geographic expansion and prospects for biological control. J Pest Sci. 2010;83: 197–215. 10.1007/s10340-010-0321-6

[9] Steck GJ. Ceratitis cosyra (Walker) (Diptera: Tephritidae). Entomol Cirular. 2000;403.

[10] Goergen G, Vayssières J-F, Gnanvossou D, Tindo M. Bactrocera invadens (Diptera: Tephritidae), a New Invasive Fruit Fly Pest for the Afrotropical Region: Host Plant Range and Distribution in West and Central Africa. Environ Entomol. 2011;40: 844–854. 10.1603/EN11017 22251685

[11] Rosenzweig CE, Iglesiaas A, Yang XB, Epstein RP, Chivian E. Climate change and extreme weather events: Implications for food production, plant diseases, and pests. Global Change Human Health. Glob Change Hum Health. 2001;2: 90–104.

[12] Coakley SM, Scherm H, Chakraborty S. CLIMATE CHANGE AND PLANT DISEASE MANAGEMENT. Annu Rev Phytopathol. 1999;37: 399–426. 10.1146/annurev.phyto.37.1.399 11701829

[13] Jaramillo J, Muchugu E, Vega FE, Davis A, Borgemeister C, Chabi-Olaye A. Some Like It Hot: The Influence and Implications of Climate Change on Coffee Berry Borer (Hypothenemus hampei) and Coffee Production in East Africa. Thrush S, editor. PLoS ONE. 2011;6: e24528 10.1371/journal.pone.0024528 21935419 PMC3173381

[14] Jaramillo J, Chabi-Olaye A, Kamonjo C, Jaramillo A, Vega FE, Poehling H-M, et al Thermal Tolerance of the Coffee Berry Borer Hypothenemus hampei: Predictions of Climate Change Impact on a Tropical Insect Pest. Rands S, editor. PLoS ONE. 2009;4: e6487 10.1371/journal.pone.0006487 19649255 PMC2715104

[15] Ward NL, Masters GJ. Linking climate change and species invasion: an illustration using insect herbivores. Glob Change Biol. 2007;13: 1605–1615.

[16] Netherer S, Schopf A. Potential effects of climate change on insect herbivores in European forests—General aspects and the pine processionary moth as specific example. For Ecol Manag. 2010;259: 831–838. 10.1016/j.foreco.2009.07.034

[17] Harrington R, Fleming RA, Woiwod IP. Climate change impacts on insect management and conservation in temperate regions: can they be predicted? Agric For Entomol. 2001;3: 233–240. 10.1046/j.1461-9555.2001.00120.x

[18] Walther G-R, Roques A, Hulme PE, Sykes MT, Pyšek P, Kühn I, et al Alien species in a warmer world: risks and opportunities. Trends Ecol Evol. 2009;24: 686–693. 10.1016/j.tree.2009.06.008 19712994

[19] Kroschel J, Sporleder M, Tonnang HEZ, Juarez H, Carhuapoma P, Gonzales JC, et al Predicting climate-change-caused changes in global temperature on potato tuber moth Phthorimaea operculella (Zeller) distribution and abundance using phenology modeling and GIS mapping. Agric For Meteorol. 2013;170: 228–241. 10.1016/j.agrformet.2012.06.017

[20] Dillon ME, Wang G, Huey RB. Global metabolic impacts of recent climate warming. Nature. 2010;467: 704–706. 10.1038/nature09407 20930843

[21] Deutsch CA, Tewksbury JJ, Huey RB, Sheldon KS, Ghalambor CK, Haak DC, et al Impacts of climate warming on terrestrial ectotherms across latitude. Proc Natl Acad Sci. 2008;105: 6668–6672. 10.1073/pnas.0709472105 18458348 PMC2373333

[22] Baini L, Ma J, Hu X, Liu H, Zhang R. Potential Geographical Distributions of the Fruit Flies Ceratitis capitata, Ceratitis cosyra, and Ceratitis rosa in China. J Econ Entomol. 2009;102: 1781–1790. 10.1603/029.102.0508 19886442

[23] Kumar S, Graham J, West AM, Evangelista PH. Using district-level occurrences in MaxEnt for predicting the invasion potential of an exotic insect pest in India. Comput Electron Agric. 2014;103: 55–62. 10.1016/j.compag.2014.02.007

[24] Solhjouy-Fard S, Sarafrazi A, Moeini MM, Ahadiyat A. Predicting habitat distribution of five heteropteran pest species in Iran. J Insect Sci. 2013;13: 116 10.1673/031.013.11601 24735397 PMC4011372

[25] Kumar S, Neven LG, Yee WL. Assessing the Potential for Establishment of Western Cherry Fruit Fly Using Ecological Niche Modeling. J Econ Entomol. 2014;107: 1032–1044. 10.1603/EC14052 25026662

[26] Sobek-Swant S, Kluza DA, Cuddington K, Lyons DB. Potential distribution of emerald ash borer: What can we learn from ecological niche models using Maxent and GARP? For Ecol Manag. 2012;281: 23–31. 10.1016/j.foreco.2012.06.017

[27] Barredo JI, Strona G, de Rigo D, Caudullo G, Stancanelli G, San-Miguel-Ayanz J. Assessing the potential distribution of insect pests: case studies on large pine weevil (*Hylobius abietis* L) and horse-chestnut leaf miner (*Cameraria ohridella*) under present and future climate conditions in European forests. EPPO Bull. 2015;45: 273–281. 10.1111/epp.12208

[28] Kumar S, Neven LG, Zhu H, Zhang R. Assessing the Global Risk of Establishment of Cydia pomonella (Lepidoptera: Tortricidae) using CLIMEX and MaxEnt Niche Models. J Econ Entomol. 2015; tov166. 10.1093/jee/tov166 26470312

[29] Baini Li, Wei Wu, Ma Jun, Zhang RunJie. Maximum entropy niche-based modeling (Maxent) of potential geographical distributions of fruit flies Dacus bivittatus, D. ciliatus and D. vertebrates (Diptera: Tephritidae). Acta Entomol Sin. 2009;52: 1122–1131.

[30] Tonnang HEZ, Mohamed SF, Khamis F, Ekesi S. Identification and Risk Assessment for Worldwide Invasion and Spread of Tuta absoluta with a Focus on Sub-Saharan Africa: Implications for Phytosanitary Measures and Management. Guedes RNC, editor. PLOS ONE. 2015;10: e0135283 10.1371/journal.pone.0135283 26252204 PMC4529269

[31] De Meyer M, Robertson MP, Mansell MW, Ekesi S, Tsuruta K, Mwaiko W, et al Ecological niche and potential geographic distribution of the invasive fruit fly Bactrocera invadens (Diptera, Tephritidae). Bull Entomol Res. 2010;100: 35 10.1017/S0007485309006713 19323851

[32] Olwoch JM, Van Jaarsveld AS, Scholtz CH, Horak IG. Climate change and the genus Rhipicephalus (Acari: Ixodidae) in Africa. Onderstepoort J Vet Res. 2007;74: 45–72. 17708153 10.4102/ojvr.v74i1.139

[33] Gregory PJ, Johnson SN, Newton AC, Ingram JSI. Integrating pests and pathogens into the climate change/food security debate. J Exp Bot. 2009;60: 2827–2838. 10.1093/jxb/erp080 19380424

[34] Müller C, Cramer W, Hare WL, Lotze-Campen H. Climate change risks for African agriculture. Proc Natl Acad Sci. 2011;108: 4313–4315. 10.1073/pnas.1015078108 21368199 PMC3060257

[35] GBIF Data Portal [Internet]. 2015. Available: www.gbif.org

[36] EMBL. Geographically tagged INSDC sequences European Molecular Biology Laboratory (EMBL); 2014.

[37] Gacemi A, Guenaoui Y. Efficacy of Emamectin Benzoate on Tuta absoluta Meyrick (Lepidoptera: Gelechiidae) Infesting a Protected Tomato Crop in Algeria. Acad J Entomol. 2012;5: 37–40.

[38] Baldé A. Detection Of Tuta absoluta (Meyrick, 1917) in Cape Verde. 2013.

[39] Russell IPM L. Tuta absoluta information network [Internet]. 2015 [cited 22 Jul 2015]. Available: www.tutaabsoluta.com

[40] Bettaibi A, Mezghani-Khemakhem M, Bouktila D, Makni H, Makni M. Genetic Variability of the Tomato Leaf Miner (Tuta absoluta Meyrick; Lepidoptera: Gelechiidae), in Tunisia, inferred from RAPD-PCR. Chil J Agric Res. 2012;72: 212–216.

[41] Royal Museum of Central Africa. True Fruit Flies (Diptera, Tephritidae) of the Afrotropical Region (ENBI wp13). Royal Museum of Central Africa; 2014.

[42] Nboyine JA, Billah MK, Afreh-Nuamah K. Species range of fruit flies associated with mango from three agro-ecological zones in Ghana. 2012; Available: http://ugspace.ug.edu.gh/handle/123456789/2393

[43] Fadlelmula AA, Eltayeb BMA, Eltayeb B. Fruit Fly Species, Their Distribution, Host Range and Seasonal Abundance in Blue Nile State, Sudan. Persian Gulf Crop Prot. 2014;3.3: 17–24.

[44] Hijmans RJ, Cameron SE, Parra JL, Jones PG, Jarvis A. Very High Resolution Interpolated Climate Surfaces for Global Land Areas. Int J Climatol. 2005;25: 1965–1978.

[45] Hijmans RJ, Cameron SE, Parra JL, Jones PG, Jarvis A. WORLDCLIM—a set of global climate layers (climate grids) [Internet]. Available: www.worldclim.com

[46] Sylla MB, Diallo I, Pal JS. West African Monsoon in State-of-the-Science Regional Climate Models In: Tarhule A, editor. Climate Variability—Regional and Thematic Patterns. InTech; 2013 Available: http://www.intechopen.com/books/climate-variability-regional-and-thematic-patterns/west-african-monsoon-in-state-of-the-science-regional-climate-models

[47] Gbobaniyi E, Sarr A, Sylla MB, Diallo I, Lennard C, Dosio A, et al Climatology, annual cycle and interannual variability of precipitation and temperature in CORDEX simulations over West Africa: CORDEX SIMULATIONS OVER WEST AFRICA. Int J Climatol. 2014;34: 2241–2257. 10.1002/joc.3834

[48] Nikulin G, Jones C, Giorgi F, Asrar G, Büchner M, Cerezo-Mota R, et al Precipitation Climatology in an Ensemble of CORDEX-Africa Regional Climate Simulations. J Clim. 2012;25: 6057–6078. 10.1175/JCLI-D-11-00375.1

[49] Elith J, H. Graham C, P. Anderson R, Dudík M, Ferrier S, Guisan A, et al Novel methods improve prediction of species’ distributions from occurrence data. Ecography. 2006;29: 129–151. 10.1111/j.2006.0906-7590.04596.x

[50] Phillips SJ, Anderson RP, Schapire RE. Maximum entropy modeling of species geographic distributions. Ecol Model. 2006;190: 231–259. 10.1016/j.ecolmodel.2005.03.026

[51] Wand MP. Fast Computation of Multivariate Kernel Estimators. J Comput Graph Stat. 1994;3: 433–445. 10.1080/10618600.1994.10474656

[52] Liu C, Berry PM, Dawson TP, Pearson RG. Selecting thresholds of occurrence in the prediction of species distributions. Ecography. 2005;28: 385–393. 10.1111/j.0906-7590.2005.03957.x

[53] Manel S, Williams HC, Ormerod SJ. Evaluating presence-absence models in ecology: the need to account for prevalence: Presence-absence modelling. J Appl Ecol. 2002;38: 921–931. 10.1046/j.1365-2664.2001.00647.x

[54] McPHERSON JM, Jetz W, Rogers DJ. The effects of species’ range sizes on the accuracy of distribution models: ecological phenomenon or statistical artefact?: Species’ range and distribution model accuracy. J Appl Ecol. 2004;41: 811–823. 10.1111/j.0021-8901.2004.00943.x

[55] Ramankutty N, Evan AT, Monfreda C, Foley JA. Farming the planet: 1. Geographic distribution of global agricultural lands in the year 2000: GLOBAL AGRICULTURAL LANDS IN 2000. Glob Biogeochem Cycles. 2008;22: n/a–n/a. 10.1029/2007GB002952

[56] Knox J, Hess T, Daccache A, Wheeler T. Climate change impacts on crop productivity in Africa and South Asia. Environ Res Lett. 2012;7: 034032 10.1088/1748-9326/7/3/034032

[57] Wheeler T, von Braun J. Climate Change Impacts on Global Food Security. Science. 2013;341: 508–513. 10.1126/science.1239402 23908229

[58] Müller C. African Lessons on Climate Change Risks for Agriculture. Annu Rev Nutr. 2013;33: 395–411. 10.1146/annurev-nutr-071812-161121 23528178

[59] Vermeulen SJ, Aggarwal PK, Ainslie A, Angelone C, Campbell BM, Challinor AJ, et al Options for support to agriculture and food security under climate change. Environ Sci Policy. 2012;15: 136–144. 10.1016/j.envsci.2011.09.003

[60] Townsend Peterson A, Papeş M, Eaton M. Transferability and model evaluation in ecological niche modeling: a comparison of GARP and Maxent. Ecography. 2007;30: 550–560. 10.1111/j.0906-7590.2007.05102.x

[61] Heikkinen RK, Luoto M, Araújo MB, Virkkala R, Thuiller W, Sykes MT. Methods and uncertainties in bioclimatic envelope modelling under climate change. Prog Phys Geogr. 751;30: 2006.

[62] Pulliam HR. On the relationship between niche and distribution. Ecol Lett. 2000;3: 349–361. 10.1046/j.1461-0248.2000.00143.x

[63] Guisan A, Thuiller W. Predicting species distribution: offering more than simple habitat models. Ecol Lett. 2005;8: 993–1009. 10.1111/j.1461-0248.2005.00792.x 34517687

