# Supplementary material for: Future Risks of Pest Species under Changing Climatic Conditions
Source: PLoS One. 2016 Apr 7;11(4):e0153237. doi: 10.1371/journal.pone.0153237 (PMC4824351; doi:10.1371/journal.pone.0153237)
Supplement: S3 Fig — Modelled habitat suitability for Tuta absoluta for current climatic conditions (a) and future climatic conditions (b—e) as mean values over suitability modelled from bioclimatic data for 3 different Generalized Circulation Models (GCM) under each representative concentration pathways (RCP) scenario as well as mean change over all RCP scenarios. (PDF) [file pone.0153237.s003.pdf]

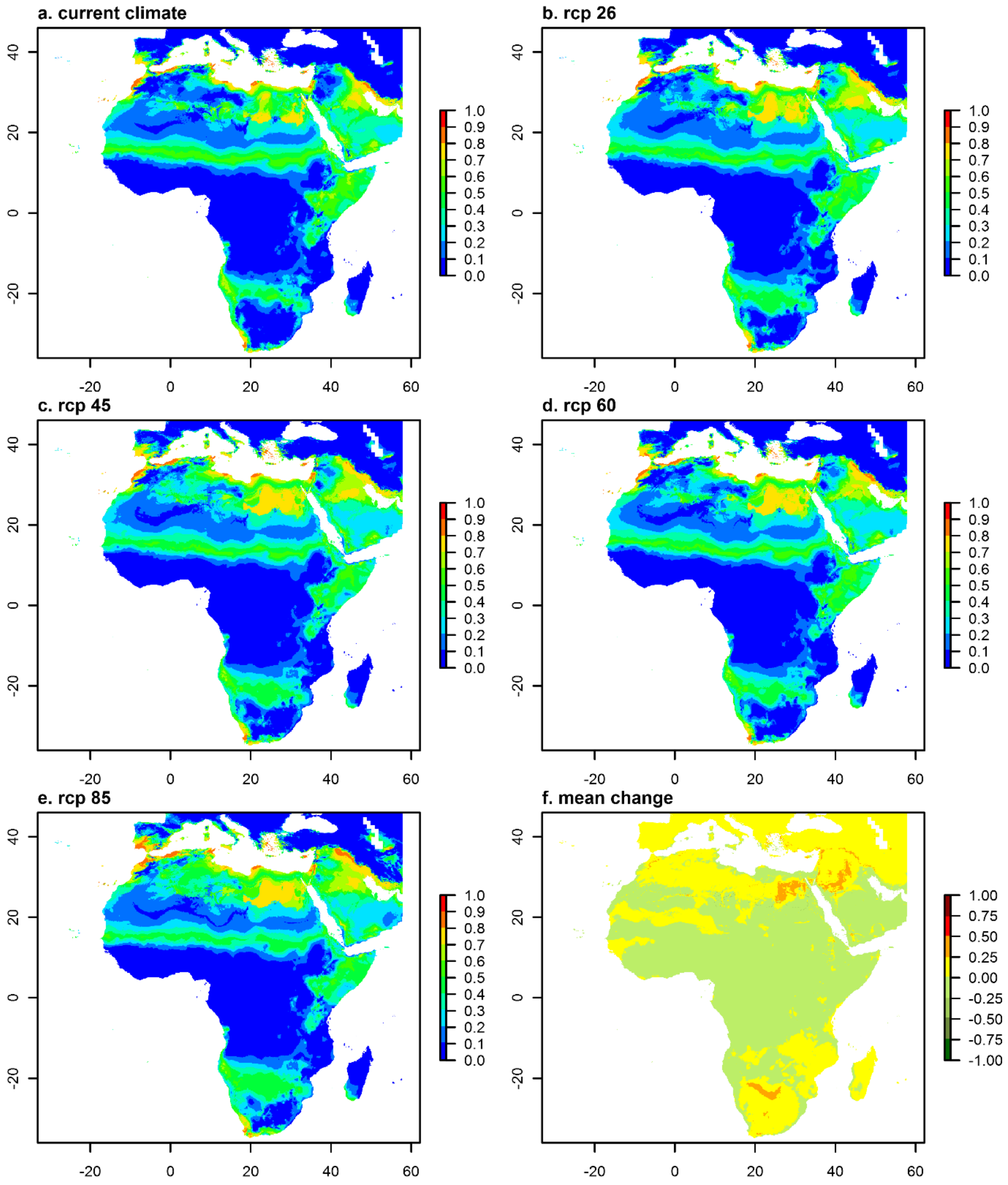

S3 Fig: Modelled habitat suitability for *Tuta absoluta* for current climatic conditions (a) and future climatic conditions (b - e) as mean values over suitability modelled from bioclimatic data for 3 different Generalized Circulation Models (GCM) under each representative concentration pathways (RCP) scenario as well as mean change over all RCP scenarios.
