# Supplementary material for: Future Risks of Pest Species under Changing Climatic Conditions
Source: PLoS One. 2016 Apr 7;11(4):e0153237. doi: 10.1371/journal.pone.0153237 (PMC4824351; doi:10.1371/journal.pone.0153237)
Supplement: S4 Fig — Response curves show effect of each variable in isolation on the model which makes interpretation of strongly correlated variables easier. (PDF) [file pone.0153237.s004.pdf]

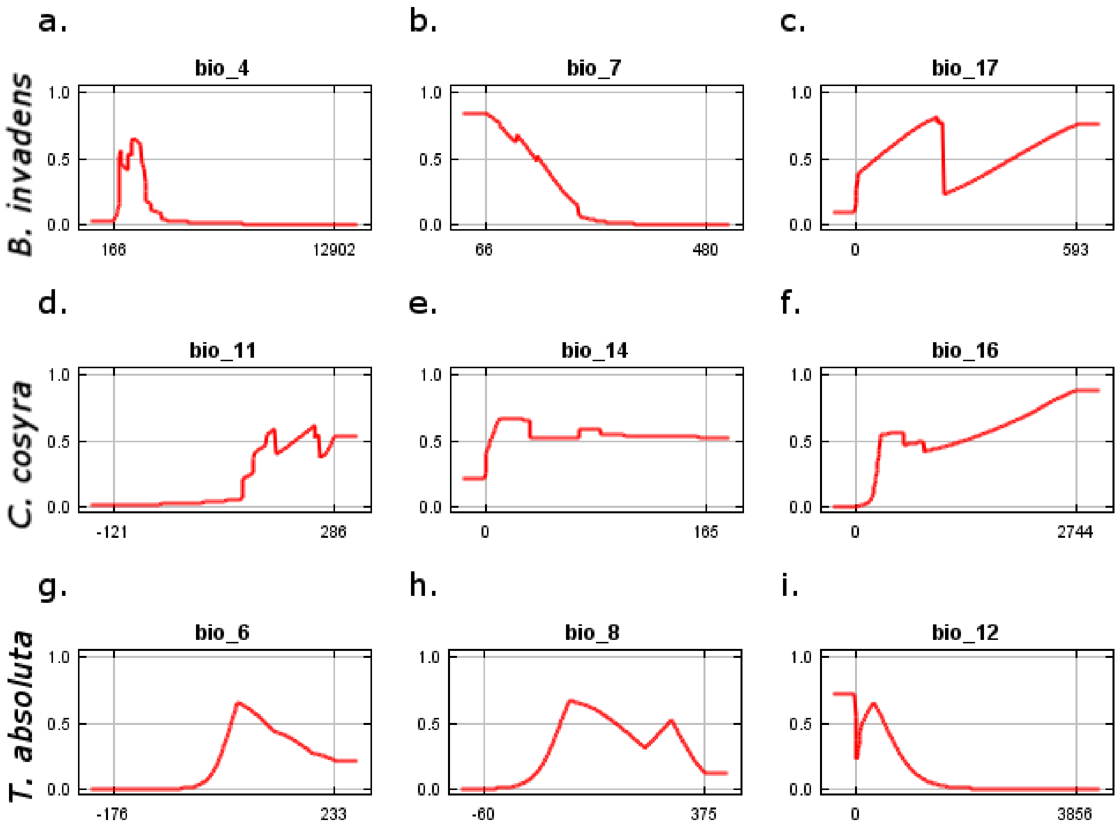

S4 Fig: Response curves of environmental variables indicating their effect on the predicted habitat suitability of Maxent with the logistic model output on the y-axis and the environmental variable on the x-axis. Response curves show effect of each variable in isolation on the model which makes interpretation of strongly correlated variables easier.
